# A nationwide study of community- versus facility-based management of malaria in Sierra Leone from 2019 to 2023

**DOI:** 10.5588/pha.25.0050

**Published:** 2026-08-24

**Authors:** M. Sillah-Kanu, D. Kamara, I.F. Kamara, B.D. Fofanah, O.O. Fiona, A.O. Vandy, A.R.Y. Kamara, W.K. Lahai, H. Owusu, R. Zachariah, A. Ramsay, A.M. Falama, M.A. Sesay, S. Lakoh, M.S. Kanu, Y.S. Tejan, I. SorieTuray, F. Thomas, J.S. Squire

**Affiliations:** 1 National Malaria Control Programme, Ministry of Health, Freetown, Sierra Leone;; 2 Directorate of Environmental Sanitation, Ministry of Water Resources and Sanitation, Freetown, Sierra Leone;; 3 World Health Organization, Sierra Leone Country Office, Freetown, Sierra Leone;; 4 Korle Bu Teaching Hospital, Accra, Ghana;; 5 UNICEF, UNDP, World Bank, WHO Special Programme for Research and Training in Tropical Diseases (TDR), Geneva, Switzerland;; 6 Infection and Global Health Division, School of Medicine, University of St Andrews, Scotland, UK;; 7 Directorate of Disease Prevention and Control, Ministry of Health, Freetown, Sierra Leone;; 8 Informatics Consultancy Firm, Freetown, Sierra Leone;; 9 Port Loko Regional Hospital, Ministry of Health, Port Loko, Sierra Leone;; 10 Directorate of Policy Planning and Information, Ministry of Health, Freetown, Sierra Leone;; 11 Department of Pharmacovigilance and Clinical trials, Pharmacy Board of Sierra Leone, Freetown, Sierra Leone;; 12 National Public Health Agency, Freetown, Sierra Leone.

**Keywords:** malaria case management, community health workers, health facilities, timely treatment

## Abstract

**SETTING:**

A nationwide analysis in Sierra Leone using routine DHIS2 surveillance data, 2019–2023.

**OBJECTIVES:**

To compare community-based and health facility–based malaria case management among people presenting with fever in Sierra Leone from 2019 to 2023, in terms of 1) suspected malaria cases, 2) testing by microscopy or rapid diagnostic tests, and 3) timely initiation of artemisinin-based combination therapy (ACT) within 24 h of reported fever onset, stratified by service level and age.

**METHOD:**

Observational, cross-sectional, comparative analysis of routinely collected national surveillance data.

**RESULTS:**

A total of 16.4 million fever cases were reported, of which 90% were managed at health facilities and 10% by community health workers. Overall, 15.8 million cases were tested for malaria, and 10.0 million were confirmed. Timely initiation of ACT within 24 h of reported fever onset was higher among community-managed cases (72.1%) compared with facility-managed cases (61.8%) (risk ratio 1.7, 95% confidence interval: 1.16–1.19), including children under 5 years.

**CONCLUSION:**

Community-based malaria case management was associated with higher reported timeliness of ACT initiation compared with facility-based care. However, these findings should be interpreted cautiously, given that it is an observational design and the reliance on routinely collected surveillance data.

Malaria remains one of the most significant global public health challenges, with an estimated 249 million cases and 608,000 deaths worldwide in 2022.^1^ Despite decades of progress, the disease continues to be endemic in 83 countries, driven by fragile health systems, climatic conditions, and emerging drug and insecticide resistance.^2^ Children under 5 years of age are disproportionately affected, accounting for majority of malaria deaths globally.^1^ Global strategies emphasise universal access to diagnostic testing, either microscopy or rapid diagnostic tests (RDTs); and prompt treatment with artemisinin-based combination therapy (ACT) for confirmed cases.^3^ However, humanitarian crises, weak health systems, and disruptions in supply chains continue to undermine progress,^4^ while the COVID-19 pandemic further slowed malaria control efforts.^5^

Malaria remains the dominant cause of febrile illness and a leading contributor to morbidity and mortality in Sierra Leone. Perennial, high-intensity transmission places the entire population at risk, and children under 5 years are disproportionately at risk of severe malaria and death.^3^ Timely diagnosis and prompt treatment are central pillars of the country’s national malaria strategy. Following global guidance, there is an emphasis on testing with microscopy or RDTs and initiation of ACT for confirmed cases. However, persistent challenges in access to health facilities (HFs), diagnostic supply-chain fluctuations, and geographic barriers constrain timely case management for many communities.^4^

Community health workers (CHWs) play a key role in expanding access to malaria diagnosis and treatment through integrated community case management, particularly in settings with limited access to HFs. At the community level, CHWs conduct rapid diagnostic testing and provide ACTs for confirmed cases in line with the national malaria treatment guidelines.^5^ Evidence from multiple settings suggests that community-based platforms can increase access to malaria services; however, variability in diagnostic practices, commodity availability, supervision, and reporting quality limits generalisability of findings across countries and programme contexts.^6^ In Sierra Leone, recent assessments have documented temporal variability in RDT availability and testing rates, which complicates the interpretation of routine surveillance trends at both community and facility levels.^7^ The rationale for this nationwide assessment is three fold: First, to describe and compare patterns of malaria case management between community-based and health facility–based platforms using routinely collected national malaria data; second, to examine testing and treatment patterns by service delivery platforms, including the use of microscopy and RDTs, to inform programme planning and interpretation of surveillance data; and third, to assess the reported timeliness of ACT initiation within 24 h of fever onset across platforms and age groups, recognising that this analysis is descriptive and does not establish causal relationships.^8^ Although previous studies have examined community-based malaria case management in sub-national or experimental settings, there is limited published evidence using routine surveillance data to compare community- and facility-based malaria management at the national scale over multiple years.^9^

This study, therefore, analyses the management of suspected malaria among individuals presenting with fever at HFs and in the community by CHWs in Sierra Leone between 2019 and 2023, using aggregated routine health information system data. Findings from this analysis are intended to inform operational discussions on service delivery performance, surveillance interpretation, and future research priorities within the national malaria programme and the Ministry of Health (MoH), rather than to assess clinical outcomes or attribute effects to specific interventions.

## METHODS

This was a nationwide, comparative, cross-sectional analysis of routinely collected health information system data from 2019 to 2023.

### Setting

This study was conducted in Sierra Leone, a malaria-endemic country with perennial malaria transmission and seasonal peaks following the rainy season. All the 16 administrative districts were included in this study. Malaria services are delivered through a tiered primary health care system comprising HFs and trained CHWs providing community-based case management. Data from both service delivery points are routinely reported monthly to the national health management information system which is digitally entered into district health information system (DHIS2) platform.

### Data sources and variables

Monthly aggregate malaria data were extracted from DHIS2 platforms for the period 2019–2023. Variables included reported fever cases, malaria testing by RDT or microscopy, confirmed malaria cases, and initiation of ACT, disaggregated by service delivery platform and age group.

### Data management

Data were imported into Microsoft Excel for cleaning and preparation. Procedures included removal of duplicate records, harmonisation of reporting units, management of missing data, and standardisation of age-group categories. Derived variables included time from reported fever onset to ACT treatment initiation (less than 24 h vs. greater than 24 h), guided by DHIS2 metadata and data-element definitions.

### Study population

The study included all individuals with fever who were recorded as presenting to HFs or CHWs and reported in DHIS2 during the 5-year study period.

### Statistical analysis

Descriptive statistics were used to summarise fever cases, malaria testing, confirmed cases, and ACT treatment initiation within and after 24 h of fever onset. Timely treatment was defined as initiation of ACT within 24 h of self-reported fever onset, as recorded in DHIS2. Comparisons between community-based and health facility–based services were made using χ^2^ tests. Risk ratios (RRs) with 95% confidence intervals (CIs) were calculated. Statistical significance was defined as *P* < 0.05. Analyses were conducted using Stata version 17.

### Ethical statement

The study used anonymised, aggregate routine programme data and involved no direct patient contact. Ethical approval was obtained from the Sierra Leone Ethics and Scientific Review Committee of the Ministry of Health (SLESRC No. 020/02/2025; approved 26 February 2025) and the Union Ethics Advisory Group of the International Union Against Tuberculosis and Lung Disease. Permission to use the data was granted by the Chief Medical Officer, Ministry of Health.

## RESULTS

Between 2019 and 2023, a total of 16,436,950 fever cases were reported nationwide. Most cases were identified through HFs (14,807,385; 90%), while CHWs accounted for 1,629,565 cases (10%) (see Figure).

**FIGURE.**
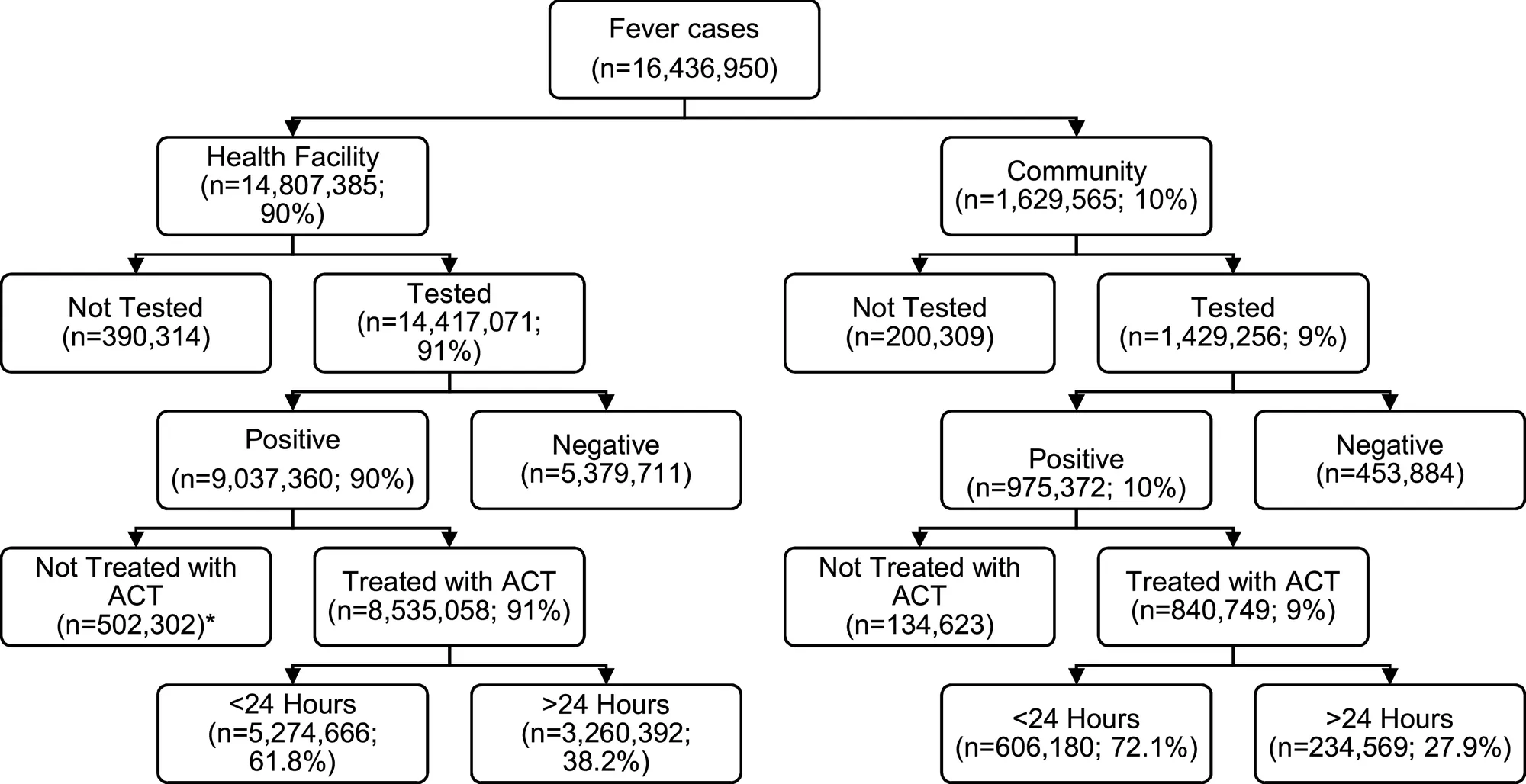
Comparing the management of malaria at health facility and community level by community health workers in Sierra Leone, 2019–2023. <24 h of onset of symptoms; >24 h of onset of symptoms. ACT = artemisinin-based combination therapy.

### Malaria testing by microscopy and RDTs

Of the 15,846,327 fever cases tested for malaria during the study period, 91% (14,417,071) were tested at HFs and 9% (1,429,256) were tested at the community level (Figure). All community testing was conducted using RDTs. At HFs, RDTs constituted 96.2% of all tests performed, while microscopy accounted for 3.8% (Table 1).

**TABLE 1.** Number and percentage of fever cases tested by microscopy and RDTs at HF and community by CHWs, 2019–2023.

Characteristic	CHW		HF		Total
	N	%^A^	N	%^A^	
Testing					
RDT	1,429,256	100.0	13,862,407	96.2	15,291,663
Microscopy	0	0.0	554,664	3.8	554,664
0–59 months	753,972	52.8	7,347,672	51	8,101,644
5–14 years	315,536	22.1	1,923,154	13.3	2,238,690
15+ years	359,748	25.2	5,146,245	35.7	5,505,993

CHW = community health worker; HF = health facility; RDT = rapid diagnostic test.

A Row percentage.

### Malaria confirmed cases

A total of 10,012,732 malaria cases were confirmed between 2019 and 2023. HFs contributed 9,037,360 confirmed cases (90%), whereas CHWs confirmed 975,372 cases (10%) (Figure).

### Treatment with ACT

Among confirmed malaria cases, 9,375,807 individuals received ACT treatment. Of these, 8,535,058 (91%) were treated at HFs, while 840,749 (9%) received treatment in the community (Figure). Timeliness of treatment differed significantly by service delivery point. Patients treated in the community were 17% more likely to receive timely ACT compared to those treated at HFs (RR = 1.17; 95% CI: 1.16–1.19; *P* < 0.001) (see Table 2).

**TABLE 2.** Timeliness of artemisinin-based combination therapy in the community and health facility by age group, 2019–2023.

Age group	Community-based (%) <24 h	Health facility (%) <24 h	Risk ratio (RR)	RR 95% CI	χ^2^ *P*
Overall	72.1	61.8	1.17	1.16–1.19	<0.001
U5 years	73.1	62.0	1.18	1.17–1.20	<0.001
5–14 years	70.9	62.2	1.14	1.13–1.15	<0.001
15+ years	71.1	61.2	1.16	1.15–1.17	<0.001

U5 years = children aged under 5 years; 5–14 years = older children aged 5–14 years; 15+ years = people 15 years and above. <24 h of symptom onset; >24 h of symptom onset. CI = confidence interval.

## DISCUSSION

This nationwide observational analysis compared malaria case management between CHWs and HFs in Sierra Leone using 5 years of routine surveillance data. Most fever cases, malaria testing, and confirmed cases were reported from HFs, while CHWs accounted for approximately one tenth of reported activity. This distribution reflects the continued central role of HFs within the health system, alongside a complementary contribution from community-based services.^10^

All malaria testing conducted by CHWs was performed using RDTs, consistent with national guidelines. HFs relied predominantly on RDTs, with a small proportion of microscopy testing. These patterns reflect differences in infrastructure and diagnostic capacity between service delivery platforms. The lower proportion of testing conducted at the community level likely reflects limited CHW coverage, variability in commodity availability, and patterns of care-seeking, rather than differences in disease burden.^4^ The reliance on RDTs by CHWs underscores their critical role in decentralising malaria diagnosis. However, the relatively low proportion of community-based testing suggests that further investment is needed to strengthen CHW networks, improve supply chain reliability, and expand coverage in hard-to-reach areas. Evidence from Sierra Leone’s National Malaria Strategic Plan emphasises that universal access to diagnostic testing is a cornerstone of malaria elimination efforts.^4^

Out of the total confirmed malaria cases, HFs again accounted for the majority (90%) and CHWs confirmed only 10%. This distribution mirrors the testing trends and highlights the centrality of HFs in malaria case management. However, the contribution of CHWs remains significant, particularly in rural and peri-urban communities where access to formal health services is limited. Policy documents and strategies have stated that CHWs can substantially increase coverage of malaria diagnosis and treatment, thereby reducing delays in care and improving outcomes.^10^ The relatively smaller share of confirmed cases at the community level may reflect both limited CHW coverage and patient preferences for facility-based care. Cultural perceptions of quality, trust in facility-based providers, and the availability of additional services at HFs may influence care-seeking behaviour.^11^

Timely initiation of ACT treatment within 24 h of reported fever onset was higher among cases managed by CHWs than those managed at HFs. This association was observed across age groups, including children under 5 years. The proximity of CHWs to households and reduced travel requirements may contribute to this observed difference. However, as this was an observational analysis using routine data, differences in case mix, severity, access to care, and reporting practices between community and facility settings could not be accounted for and may have influenced the findings.^1,12,13^ Despite the relatively higher timeliness in the community by CHWs, a substantial proportion of cases in both settings did not receive ACT within 24 h. In addition, not all fever cases were tested for malaria and not all confirmed cases received ACT treatment. These gaps highlight persistent system-level challenges, including intermittent availability of diagnostics and medicines, variability in adherence to test-and-treat guidelines, and delays in care-seeking. Similar challenges have been documented in other malaria-endemic settings using routine programmatic data.^14,15^

The policy relevance of these findings lies primarily in service delivery performance rather than health outcomes. This study did not assess clinical outcomes such as severe malaria or mortality. While community-based delivery platforms were associated with higher reported timeliness, the reasons underlying observed differences could not be determined and warrant further investigation using individual-level or mixed-methods studies. The policy implication is that, a well-trained, supervised, motivated, and equitably distributed health workforce is essential for effective programme implementation.^16^

The strengths of this study include its nationwide scope, large sample size, and use of multiple years of routine data. Limitations include reliance on aggregated surveillance data, self-reported fever onset (with potential recall bias), inability to adjust for confounding or clustering, and possible under-reporting related to diagnostic or treatment stockouts. Given the very large sample size, statistical significance should be interpreted cautiously, with greater emphasis placed on effect size and direction. To strengthen malaria case management in Sierra Leone, efforts should focus on expanding and supporting CHW coverage in hard-to-reach areas, improving supply chain to ensure uninterrupted availability of RDTs and ACTs, and reinforcing adherence to test-and-treat guidelines through targeted mentorship and supervision. Complementary individual-level and qualitative studies are needed to better understand the drivers of timeliness and care-seeking behaviour. These findings should be used by the MoH and partners for planning, optimising resource allocation, and strengthening linkages between community- and facility-based services.

## CONCLUSION

In this nationwide observational analysis, community-based malaria case management was associated with higher reported timeliness of ACT initiation compared with facility-based care. HFs, however, continued to manage most malaria cases in Sierra Leone. These findings highlight the complementary roles of community- and facility-based platforms within the national malaria programme. Further research is needed to better understand the factors influencing differences in timeliness and to assess whether these service delivery patterns translate into improved health outcomes.
